# Maternal antibiotic exposure and childhood allergies: The Japan Environment and Children’s Study

**DOI:** 10.1016/j.jacig.2023.100137

**Published:** 2023-07-06

**Authors:** Kouta Okoshi, Kenichi Sakurai, Midori Yamamoto, Chisato Mori, Japan Environment and Children's Study group, Michihiro Kamijima, Shin Yamazaki, Yukihiro Ohya, Reiko Kishi, Nobuo Yaegashi, Koichi Hashimoto, Chisato Mori, Shuichi Ito, Zentaro Yamagata, Hidekuni Inadera, Takeo Nakayama, Tomotaka Sobue, Masayuki Shima, Hiroshige Nakamura, Narufumi Suganuma, Koichi Kusuhara, Takahiko Katoh

**Affiliations:** f principal investigator, Nagoya City University, Nagoya, Japan; g National Institute for Environmental Studies, Tsukuba, Japan; h National Center for Child Health and Development, Tokyo, Japan; i Hokkaido University, Sapporo, Japan; j Tohoku University, Sendai, Japan; k Fukushima Medical University, Fukushima, Japan; l Chiba University, Chiba, Japan; m Yokohama City University, Yokohama, Japan; n University of Yamanashi, Chuo, Japan; o University of Toyama, Toyama, Japan; p Kyoto University, Kyoto, Japan; q Osaka University, Suita, Japan; r Hyogo Medical University, Nishinomiya, Japan; s Tottori University, Yonago, Japan; t Kochi University, Nankoku, Japan; u University of Occupational and Environmental Health, Kitakyushu, Japan; v Kumamoto University, Kumamoto, Japan; a Department of Sustainable Health Science, Graduate School of Medical and Pharmaceutical Sciences, Chiba University, Chiba, Japan; e Department of Bioenvironmental Medicine, Graduate School of Medicine, Chiba University, Chiba, Japan; c Department of Nutrition and Metabolic Medicine, Center for Preventive Medical Sciences, Chiba University, Japan; d Department of Sustainable Health Science, Center for Preventive Medical Sciences, Chiba University, Japan; b Innovation Center, Central Research Laboratory, NIPPN Corporation, Kanagawa, Japan

**Keywords:** Antibiotics, pregnancy, childhood allergy, preschool asthma, atopic dermatitis, food allergy, allergic rhinoconjunctivitis, birth cohort

## Abstract

**Background:**

The association of maternal antibiotic exposure during pregnancy with childhood allergic diseases remains unclear.

**Objective:**

We aimed to evaluate the association of maternal exposure to antibiotic use during pregnancy with childhood allergic diseases up to the age of 3 years by using data from a large Japanese birth cohort.

**Methods:**

We analyzed data on 78,678 pregnant women and their offspring aged 0 to 3 years. Prenatal antibiotic exposure was defined as the use of any antimicrobial agent during pregnancy. Information was collected from maternal interviews and medical record transcripts. The outcome variables in this study included preschool asthma, wheezing, food allergy, atopic dermatitis, eczema, allergic rhinoconjunctivitis, and any allergic disease. We used logistic regression analysis to evaluate the association of antibiotic exposure during pregnancy with childhood allergic diseases.

**Results:**

Among the participating mothers, 28.5% used antibiotics during pregnancy. Antibiotic exposure during pregnancy was associated with preschool asthma (adjusted odds ratio [aOR] = 1.12 [95% CI = 1.06-1.19]), wheezing (aOR = 1.11 [95% CI = 1.07-1.15]), allergic rhinoconjunctivitis (aOR = 1.10 [95% CI = 1.03-1.17]) and any allergic disease (aOR = 1.09 [95% CI = 1.05-1.14]) in offspring up to age 3 years. In contrast, maternal antibiotic use was not associated with food allergies, atopic dermatitis, or eczema. Additionally, the significant associations were not influenced by the timing of antibiotic exposure, sex of the infants, or maternal history of allergies.

**Conclusion:**

Maternal antibiotic exposure during pregnancy is associated with an increased risk of childhood respiratory allergies.

The prevalence of childhood allergic diseases such as asthma, atopic dermatitis (AD), food allergy (FA), and allergic rhinoconjunctivitis has increased significantly over the past few decades.^1^^,^^2^ A Japanese birth cohort study showed that the prevalences of asthma, AD, FA, and allergic rhinoconjunctivitis at age 3 years were 4.5%, 6%, 5.2%, and 4.5%, respectively.^3^ These allergies are common chronic inflammatory diseases, but they often coexist with other comorbidities such as obesity, sleep disorders, cognitive disorders, and mental problems, which reduce quality of life.^4^^,^^5^ Furthermore, chronic allergic diseases result in heavy socioeconomic burdens on families and countries because of high medical costs, school absenteeism, and parental absence at work.^6^

The microbiome in early life is an important factor in the development of diseases, including allergic diseases such as asthma and AD, in children,.^7^^,^^8^ Infancy is a critical period during which the microbiome develops, affecting the acquisition of microbial flora diversity and development of the immune system.^9^^,^^10^ Environmental factors in early life such as mode of delivery, breast-feeding, pet ownership, and exposure to antibiotics affect the microbiome and are associated with allergic diseases.^11^

Antibiotics play an important role in the prevention and treatment of bacterial infections, with 20% to 40% of the population using antibiotics during pregnancy and childbirth.^12^, ^13^, ^14^ However, maternal antibiotic use during pregnancy affects the vaginal microbiota, which could prevent the subsequent transfer of microbes to the baby during delivery.^15^^,^^16^ Additionally, the maternal microbiome during pregnancy plays an important role in development of the offspring's innate immune system.^17^ A systematic review investigating the risk of childhood asthma as a result of antibiotic exposure during pregnancy showed significant relationships in many studies; however, some studies showed inconsistent findings.^18^ Furthermore, in some studies, the association of prenatal antibiotic exposure with allergies in offspring varied depending on the timing of antibiotic exposure during pregnancy and maternal history of allergies.^19^^,^^20^ Moreover, there are few reports on the relationship between antibiotic exposure during pregnancy and general allergic disorders such as AD, FA, and allergic rhinoconjunctivitis.^18^^,^^20^^,^^21^ Therefore, the association of maternal antibiotic use during pregnancy with risk of childhood allergic diseases is not well established. In this study, we aimed to evaluate the association of antibiotic exposure during pregnancy with the risk of allergic diseases in children up to age 3 years by using data from a large Japanese birth cohort. Additionally, we evaluated the effect of the timing of antibiotic exposure, sex of the infants, and maternal history of allergies on the risk of development of allergic diseases during childhood.

## Methods

### Study population

The Japan Environment and Children’s Study (JECS) is a nationwide government-funded birth cohort study; its design and baseline profile have been described previously.^22^^,^^23^ The JECS cohort recruited expectant mothers from 15 regional centers in Japan from January 2011 to March 2014, enrolling 103,060 pregnancies. The present study is based on the data set of jecs-ta-20190930, which was released in October 2019. For this study, the data collected comprised antibiotic use as the exposure and allergic diseases in offspring up to age 3 years as the outcome.

**Fig 1:**
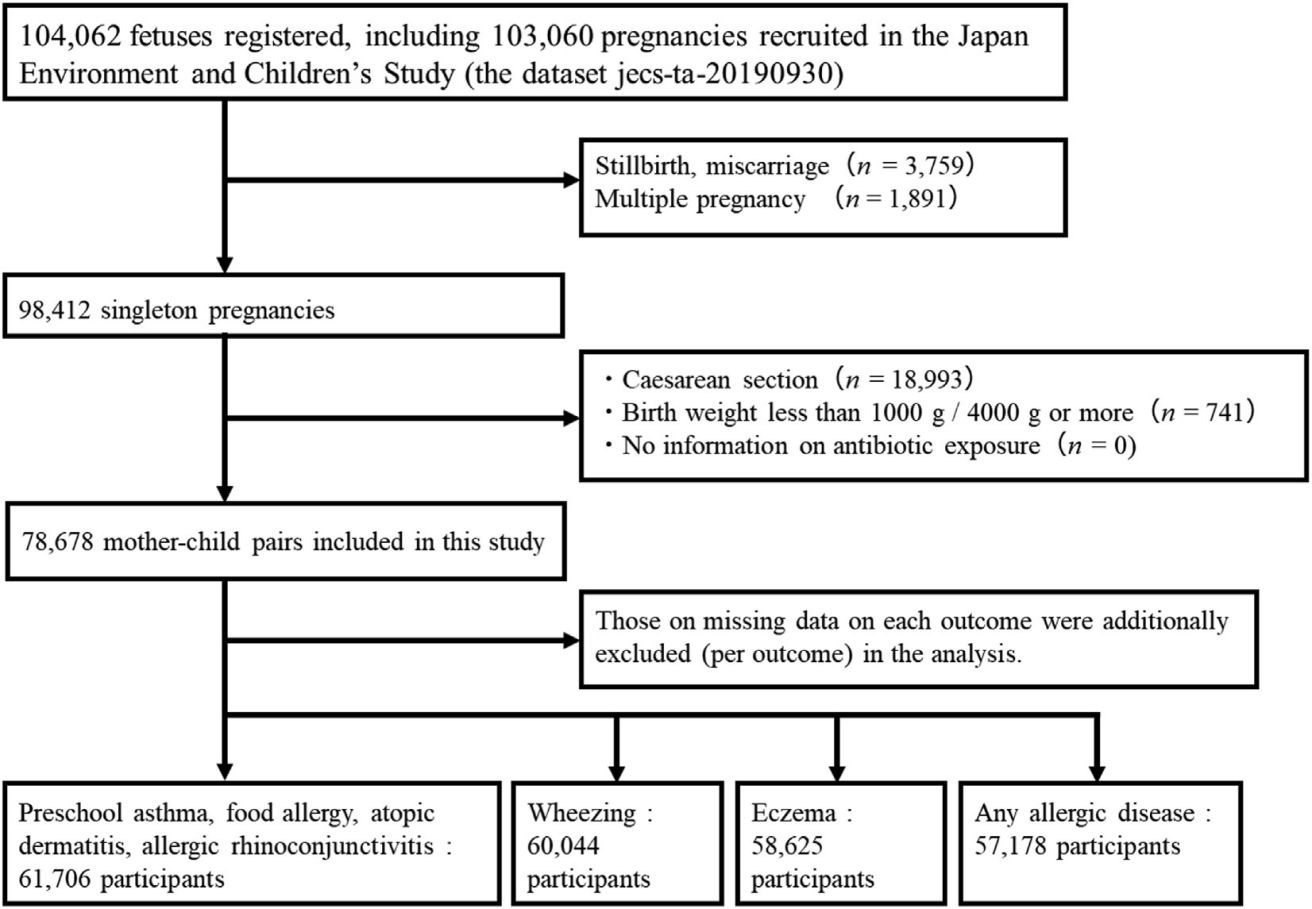
Flowchart showing selection of the study population.

In this study, 98,412 singleton pregnancies were included. The exclusion criteria were birth by caesarean section, extremely high (≥4000 g) or low (<1000 g) birth weight, and absence of information about antibiotic use. Among the remaining 78,678 mothers, those with missing data on each outcome were additionally excluded (per outcome) from the analysis (Fig 1).

### Ethics

The JECS protocol was reviewed and approved by the Ministry of the Environment's Institutional Review Board on Epidemiologic Studies and the ethics committees of all of the participating institutions. The JECS was conducted in accordance with the Declaration of Helsinki and other nationally valid regulations and guidelines. Written informed consent was obtained from all participants.

### Antibiotic exposure

Information on prenatal antibiotic exposure, which was defined as use of an antimicrobial agent during pregnancy, was collected by (1) interview questionnaire answered by the mothers at enrollment and during mid-to-late pregnancy and (2) review of the medical record transcripts that were developed after delivery. Regarding the interview questionnaires, answers about medication use were recorded by Research Co-ordinators according to a medication list. This study used only data on antibiotic exposure after the confirmation of pregnancy. For the review of medical record transcripts, exposure to antibiotics was designated when the word *antibiotics* or any antibiotic name was described as a medication, except for topical antibiotics such as ointments, gels, or drops. Antibiotic administration before caesarean section was not recorded. We combined data from the interview questionnaires and medical record transcripts to define antibiotic exposure during pregnancy.

The timing of antibiotic exposure was divided into the first (T1) and second/third (T2/3) trimester according to the interview questionnaire. The interview questionnaires used in this study included questions such as, “Did you take any antibiotics between the confirmation of pregnancy and gestational age of 12 weeks?” and “Did you take any antibiotics from after gestational age of 12 weeks until now?” We analyzed the effects of the timing of antibiotic exposure by using this classification.

### Allergic outcomes

Information on allergic outcomes was obtained from questionnaires administered when the offspring were aged 1, 1.5, 2, or 3 years. The outcome variables in this study included preschool asthma, wheezing, FA, AD, eczema, allergic rhinoconjunctivitis, and any allergic disease. The prevalence of allergic diseases (preschool asthma, FA, AD, and allergic rhinoconjunctivitis) was assessed on the basis of a caregiver-reported doctor’s diagnosis from the questionnaires at each age. Symptoms of wheezing and eczema were assessed by using a modified Japanese-translated version of the International Study of Asthma and Allergies in Childhood (ISAAC),^24^, ^25^, ^26^ which was validated on the basis of the ISAAC protocol for children aged 6 to 7 years, modified for age at the time of assessment. Wheezing was defined as having wheezing or whistling in the chest in the past 12 months. Similarly, eczema was defined as having an itchy skin rash that recurred on specific locations for at least 2 months (see Table E1 in the Online Repository at www.jaci-global.org). “Any allergic disease” was defined as the presence of any allergic outcome, including preschool asthma, FA, AD, allergic rhinoconjunctivitis, wheezing, and eczema. The prevalence of allergic diseases or symptoms up to age 3 years was defined as positive if it occurred at any time up to age 3 years.

### Other variables

On the basis of studies of the risk factors for childhood allergic diseases,^27^^,^^28^ the following covariates were selected and included in the multivariate analysis: maternal age at delivery, parity, marital status (married, unmarried, divorced, or in bereavement), prepregnancy body mass index, preexisting hypertension and diabetes, maternal history of allergies, antipyretic or analgesic use during pregnancy, maternal education (junior high school or high school; technical junior college, technical or vocational college, or associate degree; or bachelor’s degree or graduate degree), household income (<4 million yen, ≥4 but <6 million yen, or ≥6 million yen), obstetric complications (eg, threatened abortion, gestational diabetes, placenta previa), morning sickness (never, having nausea but no vomiting, having vomiting but being able to eat, or having vomiting and being unable to eat), weight gain during pregnancy, urinary cotinine concentration in T2/T3 (<0.31 μg/g-creatinine, 0.31 to <36.8 μg/g-creatinine, or ≥36.8 μg/g-creatinine),^29^ alcohol consumption during pregnancy (do not drink or stopped drinking before pregnancy, stopped drinking after pregnancy, or still drinking), sex of the infant, premature birth (<37 weeks), birth weight, breast-feeding (aged ≤6 months), and pet ownership (aged ≤3 years).

### Statistical analysis

Multiple logistic regression analysis was performed to estimate the odds ratios (ORs) and 95% CIs for the association of antibiotic exposure during pregnancy with allergic diseases in offspring up age 3 years. The forced entry method was used to include all covariates in estimation of the adjusted ORs (aORs). Missing data for covariates were imputed by using multiple imputation analysis by chained equations algorithm, which. was implemented with 10 sets of complete data, including the covariates. For subgroup analysis, the association of exposure to antibiotics during pregnancy with allergies in the offspring was evaluated by using the sex of the infants and maternal history of allergies as stratification factors. Additionally, we performed propensity score (PS) analysis with multiplied imputed data to reduce residual confounding. First, the individual PSs of exposure factors were calculated by logistic regression analysis. Variables for the PS analysis included all covariates used in the multivariate analyses plus the following variables that were logically assumed to be associated with the exposure and/or outcome variables: use of folic acid supplements during pregnancy, use of *Lactobacillus*-fermented beverages as supplements in T2/T3, frequency of cleaning the living room floor with a vacuum cleaner in T2/T3 (more or less than once per week), frequency of airing the futon in T2/T3 (more or less than 1 or 2 times per month), and use of air purifiers in T2/T3. Then, inverse probability of treatment weighting was applied across samples for the average treatment effect (ATE) model and model of the ATE on those treated, respectively. Finally, we estimated the ORs and 95% CIs for the association of antibiotic exposure during pregnancy with allergic disease in offspring up to age 3 years by using weighted logistic regression analysis.

All statistical analyses were performed using R, version 4.2.2 (Institute for Statistics and Mathematics, Vienna, Austria; www.r-project.org).

## Results

The characteristics of the participating mothers and their offspring are shown in Table I. Of the 78,678 mothers, 22,433 (28.5%) had used antibiotics during pregnancy. The prevalences of preschool asthma, wheezing, FA, AD, eczema, allergic rhinoconjunctivitis, and any allergic disease up to 3 years in the unexposed and exposed groups were as follows: 10.8% and 12.5%, 38.2% and 41.6%, 12.8% and 13.2%, 11.3% and 11.7%, 28.6% and 28.9%, 8.0% and 9.1%, and 59.6% and 62.5%, respectively.

**Table I:** Characteristics of the study participants

Antibiotic use during pregnancy	No		Yes	
Variables	Missing data (%)	Values	Missing data (%)	Values
Subjects, no. (%)		56,245 (71.5)		22,433 (28.5)
Maternal demographics				
Maternal age (y), mean (SD)	5 (0)	30.91 (4.94)	1 (0)	30.65 (5.11)
Parity≧1, no. (%)	1,452 (2.6)	32,710 (59.7)	446 (2)	13,073 (59.5)
Unmarried/divorced/bereaved no. (%)	1,012 (1.8)	2,353 (4.3)	298 (1.3)	1,075 (4.9)
Prepregnancy BMI (kg/m^2^), mean (SD)	39 (0.1)	20.99 (3.11)	8 (0)	21.08 (3.19)
Preexisting hypertension, no. (%)	766 (1.4)	190 (0.3)	197 (0.9)	69 (0.3)
Preexisting type 1 diabetes, no. (%)	766 (1.4)	26 (0.0)	197(0.9)	19 (0.1)
Preexisting type 2 diabetes, no. (%)	766 (1.4)	54 (0.1)	197 (0.9)	17 (0.1)
Maternal history of allergies, no. (%)	766 (1.4)	31,670 (57.1)	197 (0.9)	13,292 (59.8)
Antipyretic/analgesic use during pregnancy, no. (%)	6398 (11.4)	8,854 (17.8)	1,257(5.6)	6,560 (31.0)
Maternal education, no. (%)	1240 (2.2)		382 (1.7)	
Junior high school/high school		19,622 (35.7)		8,306 (37.7)
Technical junior college/technical or vocational college/associate degree		23,020 (41.8)		9,195 (41.7)
Bachelor’s degree/graduate degree		12,363 (22.5)		4,550 (20.6)
Household income, no. (%)	4844 (8.6)		1914 (8.5)	
<4 million yen		20,484 (39.9)		8,547 (41.7)
≥4 but <6 million yen		17,176 (33.4)		6,665 (32.5)
≥6 million yen		13,741 (26.7)		5,307 (25.9)
Complication of pregnancy or delivery, no. (%)	1,103 (2)	21,781 (39.5)	172 (0.8)	12,575 (56.5)
Morning sickness, no. (%)	1,217 (2.2)		365 (1.6)	
Never		9,560 (17.4)		3,667 (16.6)
Having nausea but no vomiting		23,650 (43.0)		9,403 (42.6)
Vomiting but being able to eat		15,843 (28.8)		6,466 (29.3)
Vomiting and being unable to eat		5,975 (10.9)		2,532 (11.5)
Weight gain during pregnancy, mean (SD)	1,307 (2.3)	10.39 (5.35)	238 (1.1)	10.38 (3.99)
Urinary cotinine concentration during pregnancy (T2/T3), no. (%)	2,035 (3.6)		722 (3.2)	
<0.31 μg/g creatinine (nonsmoker)		30,423 (56.1)		11,758 (54.2)
0.31<36.8 μg/g creatinine (passive smoker)		19,208 (35.4)		7,817 (36.0)
≥36.8 μg/g creatinine (active smoker)		4,579 (8.4)		2,136 (9.8)
Alcohol consumption during pregnancy (T2/T3), no. (%)	1,406 (2.5)		426 (1.9)	
Did not drink/stopped drinking before pregnancy		27,831 (50.8)		11,072 (50.3)
Stopped drinking after pregnancy		25,452 (46.4)		10,296 (46.8)
Still drinking, no. (%)		1,556 (2.8)		639 (2.9)
Offspring characteristics				
Male sex/female sex, no.	3 (0)	28,662/27,580 (51.0/49.0)	0 (0)	11,546/10,887 (51.5/48.5)
Premature birth, no. (%)	0 (0)	1,511 (2.7)	0 (0)	991 (4.4)
Birth weight (g), mean (SD)	0 (0)	3,058 (3.60)	0 (0)	3,041 (3.77)
Breast-feeding to age ≤6 mo, no. (%)	5,117 (9.1)	49,842 (97.5)	2,255 (10.1)	19,644 (97.4)
Pet ownership to age ≤3 y, no. (%)	9,703 (17.3)	15,308 (32.9)	4,107 (18.3)	6,218 (33.9)
Allergic disease to age ≤3 y, no. (%)				
Preschool asthma	11,957 (21.3)	4,789 (10.8)	5,015 (22.4)	2,183 (12.5)
Wheezing	13,116 (23.3)	16,454 (38.2)	5,518 (24.6)	7,036 (41.6)
FA	11,957 (21.3)	5,685 (12.8)	5,015 (22.4)	2,291 (13.2)
AD	11,957 (21.3)	5,021 (11.3)	5,015 (22.4)	2,046 (11.7)
Eczema	14151 (25.2)	12,047 (28.6)	5,902 (26.3)	4,777 (28.9)
Allergic rhinoconjunctivitis	11957 (21.3)	3,565 (8.0)	5015 (22.4)	1,589 (9.1)
Any allergy disease	15169 (27)	24,498 (59.6)	6,331 (28.2)	10,059 (62.5)

*BMI,* Body mass index.

The results of univariate and multivariate analyses are shown in Table II. Antibiotic use during pregnancy was significantly associated with preschool asthma (aOR = 1.12 [95% CI = 1.06-1.19]), wheezing (aOR = 1.11 [95% CI = 1.07-1.15]), allergic rhinoconjunctivitis (aOR = 1.10 [95% CI = 1.03-1.17]) and any allergic disease (aOR = 1.09 [95% CI = 1.05-1.14]) up to age 3 years. In contrast, maternal exposure to antibiotics was not associated with FA, AD, or eczema. The crude ORs and aORs did not differ substantially.

**Table II:** Effects of antibiotic exposure during pregnancy on allergic diseases in the offspring

Factor	Crude		Adjusted	
	OR	95% CI	OR	95% CI
Doctor-diagnosed				
Preschool asthma	1.18	1.12-1.25	1.12	1.06-1.19
FA	1.03	0.98-1.08	1.02	0.97-1.08
AD	1.04	0.99-1.10	1.02	0.97-1.08
Allergic rhinoconjunctivitis	1.15	1.08-1.22	1.10	1.03-1.17
ISAAC-based				
Wheezing	1.15	1.11-1.20	1.11	1.07-1.15
Eczema	1.01	0.97-1.05	1.00	0.96-1.05
Any allergic disease	1.13	1.08-1.17	1.09	1.05-1.14

Adjusted models were adjusted for maternal age at delivery, parity, marital status, prepregnancy body mass index, preexisting hypertension, preexisting diabetes, maternal history of allergies, antipyretic or analgesic use during pregnancy, maternal education, household income, complication of pregnancy or delivery, morning sickness, weight gain during pregnancy, urinary cotinine concentration during pregnancy, alcohol consumption during pregnancy, sex of the infant, premature birth, birth weight, breast-feeding, and pet ownership.

We examined the association of antibiotic exposure with the prevalence of allergies up to age 3 years according to the timing of antibiotic exposure. The analysis of the association between allergies in the offspring and the timing of antibiotic exposure showed results similar to those observed in the main analysis (Table III).

**Table III:** Association between timing of prenatal antibiotic exposure and allergic diseases in the offspring

Factor	Before 12 weeks				After 12 weeks			
	Crude		Adjusted		Crude		Adjusted	
	OR	95% CI	OR	95% CI	OR	95% CI	OR	95% CI
Doctor-diagnosed								
Preschool asthma	1.19	1.04-1.36	1.07	0.93-1.22	1.16	1.06-1.26	1.06	0.97-1.16
FA	1.01	0.88-1.15	1.01	0.88-1.15	0.92	0.85-1.01	0.93	0.85-1.02
AD	1.15	1.01-1.32	1.11	0.97-1.27	1.00	0.91-1.09	0.97	0.88-1.06
Allergic rhinoconjunctivitis	1.40	1.21-1.61	1.29	1.12-1.49	1.18	1.07-1.30	1.11	1.00-1.22
ISAAC-based								
Wheezing	1.25	1.14-1.37	1.15	1.05-1.26	1.20	1.13-1.27	1.12	1.05-1.19
Eczema	1.01	0.91-1.12	0.99	0.89-1.09	1.01	0.94-1.07	1.00	0.93-1.07
Any allergic disease	1.17	1.06-1.29	1.10	1.00-1.22	1.17	1.10-1.25	1.12	1.05-1.19

The timing of antibiotic exposure was classified as use before or after 12 gestational age of weeks according to the interview questionnaire answered by the mothers and did not include information from the medical record transcripts. Adjusted models were adjusted for maternal age at delivery, parity, marital status, prepregnancy body mass index, preexisting hypertension, preexisting diabetes, maternal history of allergies, antipyretic or analgesic use during pregnancy, maternal education, household income, complication of pregnancy or delivery, morning sickness, weight gain during pregnancy, urinary cotinine concentration during pregnancy, alcohol consumption during pregnancy, sex of the infant, premature birth, birth weight, breast-feeding, and pet ownership.

In the subgroup analysis, logistic regression analysis according to sex was performed, revealing that the aOR for males was similar to that for females (Table IV). We subsequently investigated the effects of maternal history of allergies on the association of antibiotic exposure during pregnancy with allergies in the offspring. In mothers without a history of allergies, antibiotic exposure during pregnancy was not associated with allergic rhinoconjunctivitis in their offspring (Table V). However, preschool asthma, wheezing, and any allergic disease showed associations similar to those for the whole data set, regardless of maternal history of allergies.

**Table IV:** Effects of antibiotic exposure during pregnancy on allergic diseases in male and female offspring

Factor	Male				Female			
	Crude		Adjusted		Crude		Adjusted	
	OR	95% CI	OR	95% CI	OR	95% CI	OR	95% CI
Doctor-diagnosed								
Preschool asthma	1.18	1.10-1.27	1.12	1.04-1.20	1.18	1.09-1.29	1.12	1.03-1.22
FA	1.06	0.99-1.13	1.05	0.98-1.13	0.99	0.91-1.07	0.98	0.90-1.06
AD	1.02	0.95-1.10	1.01	0.94-1.09	1.06	0.98-1.15	1.04	0.96-1.14
Allergic rhinoconjunctivitis	1.16	1.06-1.26	1.12	1.02-1.22	1.13	1.03-1.24	1.08	0.98-1.19
ISAAC-based								
Wheezing	1.17	1.11-1.23	1.12	1.06-1.18	1.14	1.08-1.20	1.10	1.04-1.16
Eczema	1.00	0.94-1.05	0.99	0.93-1.04	1.03	0.97-1.10	1.03	0.97-1.09
Any allergic disease	1.16	1.10-1.22	1.12	1.06-1.19	1.10	1.04-1.15	1.07	1.01-1.13

Adjusted models were adjusted for maternal age at delivery, parity, marital status, prepregnancy body mass index, preexisting hypertension, preexisting diabetes, maternal history of allergies, antipyretic or analgesic use during pregnancy, maternal education, household income, complication of pregnancy or delivery, morning sickness, weight gain during pregnancy, urinary cotinine concentration during pregnancy, alcohol consumption during pregnancy, sex of the infant, premature birth, birth weight, breast-feeding, and pet ow4nership.

**Table V:** Relationship between maternal history of allergies and effects of antibiotic exposure during pregnancy on allergic diseases in the offspring

Factor	No				Yes			
	Crude		Adjusted		Crude		Adjusted	
	OR	95% CI	OR	95% CI	OR	95% CI	OR	95% CI
Doctor-diagnosed								
Preschool asthma	1.16	1.06-1.28	1.11	1.01-1.22	1.17	1.10-1.25	1.12	1.05-1.20
FA	1.00	0.91-1.10	1.00	0.91-1.10	1.02	0.96-1.09	1.03	0.96-1.10
AD	0.98	0.89-1.09	0.97	0.87-1.07	1.04	0.98-1.12	1.05	0.98-1.12
Allergic rhinoconjunctivitis	1.06	0.94-1.20	1.04	0.91-1.18	1.15	1.07-1.23	1.12	1.04-1.21
ISAAC-based								
Wheezing	1.13	1.07-1.20	1.10	1.04-1.17	1.16	1.10-1.21	1.12	1.06-1.17
Eczema	0.96	0.90-1.03	0.95	0.89-1.02	1.03	0.98-1.08	1.03	0.98-1.09
Any allergic disease	1.09	1.03-1.15	1.07	1.01-1.13	1.13	1.08-1.19	1.11	1.06-1.17

Adjusted models were adjusted for maternal age at delivery, parity, marital status, prepregnancy body mass index, preexisting hypertension, preexisting diabetes, maternal history of allergies, antipyretic or analgesic use during pregnancy, maternal education, household income, complication of pregnancy or delivery, morning sickness, weight gain during pregnancy, urinary cotinine concentration during pregnancy, alcohol consumption during pregnancy, sex of the infant, premature birth, birth weight, breast-feeding, and pet ownership.

In the PS analysis, antibiotic exposure during pregnancy was significantly associated with childhood preschool asthma, wheezing, allergic rhinoconjunctivitis, and any allergic disease, as shown by weighted logistic regression analysis in both the ATE model and the model of the ATE on those treated (see Table E2 in the Online Repository at www.jaci-global.org). In contrast, maternal exposure to antibiotics was not associated with FA, AD, or eczema. Overall, the results of the PS analysis and logistic regression analysis without PS did not differ substantially.

## Discussion

In this study, we evaluated the association of mothers' antibiotic exposure during pregnancy with the prevalence of allergies in their offspring. Our results showed that maternal antibiotic use during pregnancy was associated with an increased risk of preschool asthma, allergic rhinoconjunctivitis, wheezing, and any allergic disease up to age 3 years in offspring. In contrast, our results suggest that maternal exposure to antibiotics does not contribute to the risk of developing FA, AD, and eczema. Additionally, this association was not influenced by the timing of antibiotic exposure, sex of the infant, or maternal history of allergies, except for the risk of childhood allergic rhinoconjunctivitis in the offspring of mothers without a history of allergies.

Our results are consistent with those of other studies investigating the association of antibiotic exposure during pregnancy with childhood asthma and/or wheezing. A systematic review of 12 studies on antibiotic use during pregnancy and childhood asthma by Baron et al^18^ showed that most studies had an aOR ranging from 1.13 (95% CI = 1.02-1.24) to 3.19 (95% CI = 1.52-6.67). Additionally, Zhong et al^21^ integrated aOR into 13 studies by performing a meta-analysis and showed a statistically significant risk of development of childhood asthma and/or wheezing with the use of antibiotics in pregnant mothers (aOR = 1.29 [95% CI = 1.16-1.43]). Our results showed that the timing of antibiotic exposure (T1 and T2/T3) did not affect the incidence of childhood allergic diseases, including preschool asthma and/or wheezing. Our findings are similar to those of Bai et al,^30^ who found that the risk of asthma or wheezing was similar irrespective of the timing of antibiotic use during pregnancy. In contrast, Zhong et al^21^ reported that there was a marginally higher risk of development of asthma and wheezing following antibiotic use in T3 than in T1 or T2 of pregnancy. The reason for these inconsistent results is not clear. Other studies have suggested that there is a significant dose-response relationship between childhood allergic diseases and antibiotic use during pregnancy.^31^^,^^32^ Additionally, the type of antibiotics used during pregnancy may increase the risk of asthma in childhood.^32^ Several specific antibiotics used during pregnancy were associated with childhood asthma; the strongest association was observed for cephalosporins (aOR = 1.46 [95% CI = 1.30-1.64]).^32^ Therefore, it is necessary to consider the timing of antibiotic exposure, dose, and type of antibiotics.

The evidence regarding the association of antibiotic exposure during pregnancy with childhood AD and/or eczema is inconsistent. Similar to us, Sasaki et al^33^ found that in the JECS, antibiotic exposure during pregnancy was not associated with AD at the age of 1 year. Additionally, a cohort study conducted in the United States showed that antibiotic use during pregnancy was not associated with eczema at the age of 2 years.^34^ In contrast, a European cohort study showed a positive association between antibiotic exposure during pregnancy and childhood AD, with a significantly increased risk of diagnosed AD at the age of 1 year (aOR = 1.66 [95% CI = 1.11-2.48]).^35^ However, the risk of diagnosed AD at the age of 1.5 to 6 years in the European cohort was not significantly associated with antibiotic use during pregnancy. A Danish cohort study showed that the use of prenatal antibiotics was associated with increased odds of AD among children born to atopic mothers but only when used in both T1/T2 and T3 (aOR = 1.45 [95% CI = 1.19-1.76]).^20^

There are few findings regarding the association of antibiotic exposure during pregnancy and childhood FA. A meta-analysis by Zhong et al^21^ integrated the aORs of 3 studies and reported that antibiotic exposure was not associated with childhood FAs (aOR = 1.36 [95% CI = 0.94-1.96]); however, they did report that their findings are not conclusive owing to the small number of included studies.

Very few studies have evaluated the association between childhood allergic rhinitis and/or conjunctivitis and antibiotic exposure during pregnancy. In contrast to us, Metzler et al^35^ found that antibiotic exposure during pregnancy did not increase the risk of allergic rhinitis at age 6 years. The cause of these inconsistent results may be that previous studies had different definitions (including parental self-reporting of symptoms) of outcomes and a small number of participants. Furthermore, the finding that antibiotic exposure during pregnancy increases the risk of childhood allergic rhinoconjunctivitis in the offspring of mothers with a history of allergies is of interest for future research.

There are sex differences in the prevalence of allergic diseases, such as asthma, AD, and allergic rhinoconjunctivitis.^36^ Prepubertal girls have a lower risk of asthma and rhinitis than boys do, but these incidences become relatively balanced across the sexes after puberty.^37^ Similarly, girls have a lower risk of developing allergies by the age of 3 years than boys do (aOR for any allergic disease = 0.71 [95% CI = 0.68-0.73]), but the sex of the infants did not affect the association of antibiotic exposure during pregnancy with allergic diseases in our study. Maternal allergy is a strong determinant of allergic risk in offspring and affects fetal immune function via epigenetic regulation.^38^ This suggests that the endogenous effects of the maternal allergic phenotype may exacerbate the increasingly proallergic exogenous milieu, including antibiotic exposure during pregnancy. However, our results show that a maternal history of allergies does not affect the association of antibiotic exposure during pregnancy with childhood allergic diseases except allergic rhinoconjunctivitis. These results suggest that the mechanism by which antibiotic exposure during pregnancy increases the risk of childhood allergic diseases is unrelated or not influenced by the sex of the infants and maternal allergies.

Several confounding factors have been noted for the association between maternal antibiotic use and risk of asthma in childhood, including maternal infection-induced inflammation, vitamin D and other nutrients that affect immune regulation, and exposure to environmental chemicals^39^^,^^40^; these were not included as covariates in our study. However, the results of PS analysis suggested that the association between maternal antibiotic exposure and childhood allergy is robust. This is consistent with the association between maternal antibiotic exposure and risk of childhood allergic diseases in studies that consider infectious diseases.^41^^,^^42^

Currently, the mechanisms by which antibiotic exposure during pregnancy increases the risk of childhood allergic diseases remains unclear. However, some evidence suggests that it is mediated by the microbiome. A study of human fetuses reported that colonization of the lung microbiome occurs by the 11th week of gestation.^43^ As at least some antibiotics cross the placenta, their use during pregnancy can affect the maternal and fetal microbiome and cause changes in the gut microbiota of the offspring.^16^^,^^44^, ^45^, ^46^ Additionally, studies conducted on animal models showed that administration of antibiotics to newborns inhibits the maturation of the T_H_1 cell response and induces T_H_2 cell response by causing changes in the gut microbiota.^47^ Therefore, antibiotic exposure during pregnancy may induce allergies in the offspring by perturbing the maternal and fetal microbiome and disrupting the maturation of the immune system. A study of the nasopharyngeal microbiome in preschool children showed that children with recurrent wheezing had lower nasopharyngeal microbiome biodiversity than did children without respiratory symptoms.^48^ Furthermore, bacterial colonization of the airways in neonates is associated with subsequent childhood asthma.^49^ As previously stated, the type and dose of antibiotics used during pregnancy can affect the risk of childhood asthma.^32^ These findings support the assumption that the microbiome mediates the association of antibiotic exposure during pregnancy with childhood allergic diseases.

The strength of our study includes its large sample size. The characteristics of our study population were similar to those of the Japanese population.^22^ Additionally, the questionnaires collected from participants and doctors included variables on most of the risk factors associated with childhood allergic diseases. Therefore, our study enabled control over a large number of confounders in the model and has sufficient statistical power to accurately assess the association of antibiotic use during pregnancy with childhood allergic diseases.

The limitation of our study is that we did not have sufficient data to assess the influence of the type, dose, and administration period of the antibiotics used. Of the 22,433 cases of women who used antibiotics during pregnancy, 18,270 (81.4%) were confirmed after a review of the medical record transcripts, whereas the remaining 4,163 (18.6%) were confirmed on the basis of the mothers’ reports by using questionnaires; therefore, there may have been a risk of recall bias and misclassification. Similarly, the assessment of allergies was based on caregiver reports, which could lead to recall bias. However, for preschool asthma and AD, the results were reinforced by assessing symptoms of wheezing and eczema using the validated ISAAC questionnaire in addition to the doctor's diagnosis. Additionally, the diagnosis of preschool asthma by the age of 3 years was not definitive and may have been transient. The further development of preschool asthma toward asthma or remission cannot be indicated with the current data. However, wheezing in childhood is a predictor of future asthma, and our findings provide some clinical significance.^50^^,^^51^

In conclusion, antibiotic exposure during pregnancy was associated with an increased risk of preschool asthma, wheezing, allergic rhinoconjunctivitis, and any allergic disease up to age 3 years, but not AD, eczema, or FA. However, this finding does not preclude the use of antibiotics, as infections during pregnancy are associated with preterm labor, miscarriage, and intrauterine growth retardation.^52^^,^^53^ Considering the risks and benefits of antibiotics, it is important to use them appropriately and when necessary and to closely observe the child after birth.

## Disclosure statement

This study was funded by the 10.13039/501100006120Ministry of the Environment, Japan. The findings and conclusions of this article are solely the responsibility of the authors and do not represent the official views of the Japanese government.

Disclosure of potential conflict of interest: The authors declare that they have no relevant conflicts of interest.
