# Supplementary material for: Maternal antibiotic exposure and childhood allergies: The Japan Environment and Children’s Study
Source: J Allergy Clin Immunol Glob. 2023 Jul 6;2(4):100137. doi: 10.1016/j.jacig.2023.100137 (PMC10509907; doi:10.1016/j.jacig.2023.100137)
Supplement: Supplementary data [file mmc1.docx]

**Supplemental material**

Supplementary Table SI. Definition of wheezing and eczema

|  | Definitions |
| --- | --- |
| Wheezing | A positive answer to the question “Has your child had wheezing or whistling in the chest in the past 12 months?” |
| Eczema | (Questionnaire at the age of 1 and 1.5 years)  Positive answers to all of the following questions:  1) “Has your child ever had an itchy recurring rash for at least 2 months?”  2) “Has your child ever had an itchy rash at any time in the past 12 months?”  3) “Has this itchy rash at any time affected any of the following areas: scalp, cheek, around the eyes/ears/neck, the folds of the elbows, outer arms, wrists, thighs, inner fold of the knees, legs below the knees, or in front of the ankles?”  (Questionnaire at the age of 2 and 3 years)  Positive answers to all of the following questions:  1) “Has your child ever had an itchy recurring rash for at least 6 months?”  2) “Has your child ever had an itchy rash at any time in the past 12 months?”  3) “Has this itchy rash at any time affected any of the following areas: the folds of the elbows, inner fold of the knees, in front of the ankles, under the buttocks, or around the neck, ears or eyes?” |

Supplementary Table SII. Effects of antibiotic exposure during pregnancy on allergic disease in offspring using PS analysis

|  | ATE model | | ATT model | |
| --- | --- | --- | --- | --- |
|  | OR | 95% CI | OR | 95% CI |
| Doctor-diagnosed |  |  |  |  |
| Preschool asthma | 1.10 | 1.04–1.16 | 1.13 | 1.07–1.19 |
| Food allergy | 1.02 | 0.97–1.08 | 1.01 | 0.96–1.06 |
| Atopic dermatitis | 1.03 | 0.97–1.09 | 1.02 | 0.97–1.08 |
| Allergic rhinoconjunctivitis | 1.10 | 1.03–1.18 | 1.09 | 1.02–1.16 |
| ISAAC-based |  |  |  |  |
| Wheezing | 1.10 | 1.06–1.14 | 1.11 | 1.07–1.15 |
| Eczema | 1.01 | 0.97–1.05 | 1.01 | 0.97–1.05 |
| Any allergic disease | 1.09 | 1.05–1.13 | 1.09 | 1.05–1.13 |

PS: propensity score, ATE: average treatment effect, ATT: average treatment effect on the treated, OR: odds ratio, CI: confidence interval

OR and 95% CI were estimated by the weighted logistic regression analysis. PS was calculated by logistic regression analysis using maternal age at delivery, parity, marital status, pre-pregnancy body mass index, pre-existing hypertension, pre-existing diabetes, maternal history of allergies, antipyretics or analgesics use during pregnancy, maternal education, household income, complication of pregnancy or delivery, morning sickness, weight gain during pregnancy, urinary cotinine concentration during pregnancy, alcohol consumption during pregnancy, sex of the infant, premature birth, birth weight, breastfeeding, pet ownership, use of folic acid supplements during pregnancy, use of Lactobacillus-fermented beverages as supplements in T2/T3, frequency of cleaning the living room floor with a vacuum cleaner in T2/T3, frequency of airing the futon in T2/T3, and usage of air purifiers in T2/T3.
